# Genomic Characterization of Rift Valley Fever Virus, South Africa, 2018

**DOI:** 10.3201/eid2510.181748

**Published:** 2019-10

**Authors:** Antoinette van Schalkwyk, Marco Romito

**Affiliations:** Onderstepoort Veterinary Institute, Onderstepoort, South Africa

**Keywords:** Rift Valley fever virus, South Africa, full genomes, viruses, RVFV, Rift Valley fever, RVF

## Abstract

An isolated Rift Valley fever (RVF) outbreak was reported in 2018 in Free State Province, South Africa. Phylogenetic analyses based on complete genome sequences of 3 RVF viruses from blood and tissue samples indicated that they were related to a virus isolated in 2016 from a man returning to China from Angola.

Rift Valley fever (RVF) is endemic to sub-Saharan Africa; major outbreaks were reported in South Africa during the 1950s, the 1970s, and 2008–2011 (*1*). Molecular classification of RVF viruses (RVFVs) isolated from 16 countries showed that these viruses cluster into 15 lineages (A–O) (*2*). Viral sequences from the previous outbreaks in South Africa clustered in lineage C (2008–2009), lineage H (2009–2010), lineage I (1951), and lineage L (1974−1975); 1 isolate in 2009 from Kakamas in the Northern Cape Province was in lineage K (Figure) (*2*). Lineage K contains the hepatotropic Entebbe-44 virus isolated from mosquitoes in Uganda in 1944 and its derivative, the Smithburn neurotropic vaccine strain (SNS) commercially available in South Africa (*2*). RVFV was identified by unbiased deep sequencing of the virus genome (isolate BIME-01) from a man returning to China in 2016 with fever and jaundice after a 22-month stay in Angola (*3*). Phylogenetic analysis of the complete RVFV genome of sample BIME-01 and the Vero cell culture isolate of the virus (RVFBJ01) showed that it clustered together with the Kakamas/2009 virus in lineage K (*3*).

**Figure:**
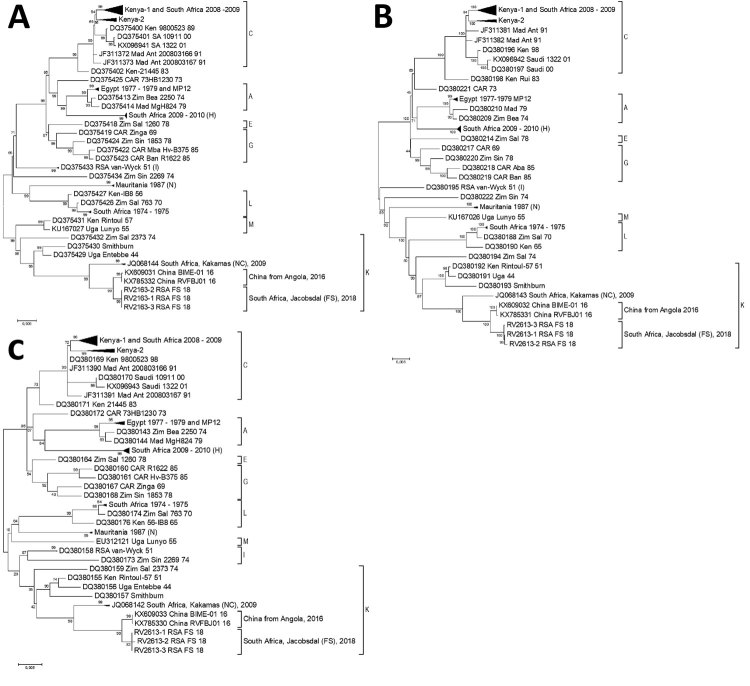
Phylogenetic comparison of the complete segments of Rift Valley fever viruses from South Africa, 2018, and reference isolates. A) Large segment; B) medium segment; C) small segment. Distinct clusters separate the isolates into 10 lineages (A, C, E, G, H, I, K, L, N, and M). Sequences RV2613/RSA/2018 are marked as South Africa, Jacobsdal (FS), 2018 in cluster K. GenBank accession numbers are provided. FS, Free State. Scale bars indicate nucleotide substitutions per site.

On April 28, 2018, an outbreak of suspected RVF was reported on a sheep farm in the Jacobsdal area of the Free State Province of South Africa. The illness rate was ≈55.8% and the case-fatality rate 100% (35 sheep died) (*4*). Six persons either working or residing on the farm reported symptoms compatible with RVFV infection, but no human fatalities occurred (*5*). Clinical specimens from affected sheep were submitted to the Ondersterpoort Veterinary Institute Agricultural Research Council (Onderstepoort, South Africa) for laboratory confirmation of the outbreak. 

We used flocculated nylon swabs (FLOQswabs, COPAN, http://www.copanusa.com) to pierce and swab the tissue pools and then placed the swabs into Eppendorf tubes containing 700 μL phosphate-buffered saline (pH 7.0). After agitation, we removed 200 μL buffer for total nucleic acid extraction. We used either whole blood in EDTA (RV2613-1/RSA/2018) or a combination of tissue swab specimens from liver, spleen, and kidney for extractions (RV2613-2/RSA/2018 and RV2613-3/RSA/2018) using the MagNA Pure 96 (Roche Molecular Systems, https://www.roche.com). We detected the presence of RVFV RNA using real-time reverse transcription PCR (RT-PCR) (*6*). We used the same 3 nucleic acid extracts as templates in 8 individual RT-PCRs (A–H), designed to overlap the entire genome (Appendix Table 1). We used the SuperScript III One-Step RT-PCR System with Platinum Taq DNA polymerase (Invitrogen, https://www.thermofisher.com) in a 20-μL reaction with 0.25 μmol/L of each primer (Appendix Table 1) at an annealing temperature of 53°C for 45 cycles. The resulting amplicons overlapped regions of all 3 genome segments: large (A, B, C, and H), medium (D, E, and F), and small (H). We submitted the 8 amplicons to Inqaba Biotechnical Industries, Pretoria, South Africa (https://www.inqababiotec.co.za), for Sanger sequencing, using the primers incorporated during the generation of the amplicons and 9 additional primers (Appendix Table 1). We constructed the complete viral genome sequences from the 3 field specimens and submitted them to GenBank (accession nos. MK134834–42).

The 3 sequences (RV2613-1/RSA/2018, RV2613-2/RSA/2018, and RV2613-3/RSA/2018) were similar to one another: no nucleotide differences in the large segment, 2 in the medium segment, and 1 in the small segment. The high sequence identity among these 3 viruses and the lack of segment reassortment, together with the isolated geographic distribution of the outbreak, indicate a single introduction. After phylogenetic analysis, we clustered the 3 viruses into lineage K, with their closest known relatives BIM-01/2016, isolated from a worker from China in Angola, and the virus RVFBJ01/2016 derived from cell culture (Figure). We assessed each genome segment and found <1% sequence difference between any of the 3 South Africa viruses and the virus from Angola and <2.11% sequence difference for Kakamas/2009 (Appendix Table 2). Evolutionary analysis of segment M using Bayesian inference with BEAST version 1.8.1 (https://beast.community) under the Hasegawa-Kishino-Yano substitution model, a strict molecular clock, and a constant population size estimated that RV2613/RSA/2018 and BIM-01/2016 had a common ancestor ≈7 years ago that shared a common ancestor with Kakamas/2009 ≈28 years ago. Virus RV2613/RSA/2018 had a higher sequence identity with the original Entebbe-44 isolate than the SNS vaccine or vaccine-derived Ken Rintoul-57 (Appendix Table 2). This result indicates that Kakamas/2009, BIM-01/2016, and RV2613/RSA/2018 probably evolved from a common ancestor of Entebbe-44 and not from its derivative SNS vaccine.

The sequence data imply that this outbreak was likely the result of a single introduction of virus that probably remained localized to 1 farm because of the onset of colder winter temperatures and a decline in rainfall. The phylogenetic relationship of this virus to known others suggests a persistent, yet largely unnoticed, low-level spread of RVFVs in southern Africa. This finding reemphasizes the importance of active disease surveillance programs with diligent reporting of suspected cases, as well as suitable vaccination regimens.

Appendix Additional information regarding genomic characterization of Rift Valley fever virus, South Africa, 2018.
