# Supplementary material for: Genomic Characterization of Rift Valley Fever Virus, South Africa, 2018
Source: Emerg Infect Dis. 2019 Oct;25(10):1979–81. doi: 10.3201/eid2510.181748 (PMC6759270; doi:10.3201/eid2510.181748)
Supplement: Appendix — Additional information regarding genomic characterization of Rift Valley fever virus, South Africa, 2018. [file 18-1748-Techapp-s1.pdf]

Article DOI: https://doi.org/10.3201/eid2510.181748

Genomic Characterization of Rift Valley Fever Virus, South Africa, 2018

Appendix

Appendix Table 1. Primers used for amplification and sequencing of the Rift Valley Fever virus genomes, South Africa, 2018.

Name	Segment, amplicon (binding site)	Sequence (5′–3′)
Primers used during amplification and sequencing		
RVF-LA-F	Large, amplicon A (1–17)	acacaaggcgcccaatc
RVF-LA-R	Large, amplicon A (2182–2163)	GGCTCACCTGCTATTCTCTG
RVF-LB-F	Large, amplicon B (2040–2059)	ATCATGGAGGGCTTTGTCTC
RVF-LB-R	Large, amplicon B (3404–3384)	CTACTATCATCTGATCCTTGC
RVF-LC-F*	Large, amplicon C (4216–4233)	ggtgttgtgtcatcattg
RVF-LC-R	Large, amplicon C (6372–6352)	acacaaagaccgcccaatattg
RVF-LH-F	Large, amplicon H (3018–3037)	GCTATGTGAGTTCACCTCTC
RVF-LH-R*	Large, amplicon H (4590–4570)	gtgtgagctagagttgcttc
RVF-MD-F	Medium, amplicon D (1–15)	acacaaagacggtgc
RVF-MD-R*	Medium, amplicon D (1342–1326)	cctgacccattagcatg
RVF-ME-F*	Medium, amplicon E (771–790)	ccaaatgactaccagtcagc
RVF-ME-R	Medium, amplicon E (2284–2264)	GATAGCTCACTTGAGACAGTT
RVF-MF-F	Medium, amplicon F (1714–1732)	ATGGCTGCATAGTGTGTGC
RVF-MF-R	Medium, amplicon F (3885–3870)	cacaaagaccggtgc
RVF-SG-F*	Small, amplicon G (1–22)	acacaaagctccctagagatac
RVF-SG-R*	Small, amplicon G (1693–1677)	acacaaagacccctagtg
Primers used only for sequencing		
RVF-LA-SF	Large, amplicon A (664–682)	AGACTGAGAGAGAGTTGCT
RVF-LA-SR	Large, amplicon A (874–855)	TCATTCCCTTCAGCAAAGAA
RVF-LC-SF	Large, amplicon C (5587–5606)	GCATCAGACAATGATCTCAG
RVF-LC-SR	Large, amplicon C (5744–5725)	tcacatatctcaaggatggc
RVF-MD-S	Medium, amplicon D (690–672)	ctttgcaatgatggtgtc
RVF-MF-SF1	Medium, amplicon F (2412–2431)	GCAGAGTTTTCATTTGTTGG
RVF-MF-SF2	Medium, amplicon F (3021–3040)	GCTTTCTCTAAGGGCTCTGT
RVF-SG-SF	Small, amplicon G (762–781)	CTCCAATCCCAGATGTTGAG
RVF-SG-SR	Small, amplicon G (1025–1007)	agccatgagaagaggagag

*Published primers (*1*).

Appendix Table 2. Sequence differences between viruses RV2613–1/RSA/2018, RV2613–2/RSA/2018 and RV2613–3/RSA/2018, and other Rift Valley fever viruses with complete genome sequences available in GenBank.*

Closest related isolates					
Isolate	Year isolated	Species; location	No. differences, segment L (%)	No. differences, segment M (%)	No. differences, segment S (%)
RV2613/RSA/2018	2018	Ovine; Jacobsdal, FS, RSA	0	2–5 nt (0.05–0.13)	1 nt(0.06)
BIM-01/2016	2016	Human; Beijing, China	30 nt (0.47)	22–26 nt (0.57–0.67)	7–8 nt(0.41–0.47)
RVFBJ01/2016	2016	Cell culture isolate of BIM-01_2016	29 nt (0.45)	24–28 nt (0.62–0.73)	7–8 nt(0.41–0.47)
Kakamas-09	2009	Ovine; Kakamas, NC, RSA	103 nt (1.61)	78–82 nt (2.01–2.11)	29–30 nt (1.72–1.78)
Entebbe-44	1944	Mosquito; Entebbe, Uganda	167 nt (2.62)	116–120 nt (2.05–2.12)	44–45 nt (2.60– 2.66)
Ken Rintoul-57	1951	Ovine; Kenya	210 nt (3.29)	129–130 nt (3.33–3.43)	40–41 nt (2.36–2.42)

*FS, Free State Province; NC, Northern Cape Province; nt: nucleotide; RSA, Republic of South Africa.

Reference

1. Grobbelaar AA, Weyer J, Leman PA, Kemp A, Paweska JT, Swanepoel R. Molecular epidemiology of Rift Valley fever virus. Emerg Infect Dis. 2011;17:2270–6. https://doi.org/10.3201/eid1712.111035
